# Cyclical Etidronate Reduces the Progression of Arterial Calcifications in Patients with Pseudoxanthoma Elasticum: A 6-Year Prospective Observational Study

**DOI:** 10.3390/jcm13164612

**Published:** 2024-08-07

**Authors:** Iris M. Harmsen, Tim van den Beukel, Madeleine Kok, Frank L. J. Visseren, Pim A. de Jong, Socrates E. Papapoulos, Wilko Spiering

**Affiliations:** 1 Department of Vascular Medicine, University Medical Center Utrecht, Utrecht University, 3508 GA Utrecht, The Netherlands; 2 Department of Radiology, University Medical Center Utrecht, Utrecht University, 3584 CX Utrecht, The Netherlands; 3 Department of Radiology & Nuclear Medicine, Rijnstate, 6815 AD Arnhem, The Netherlands; 4 Center for Bone Quality, Department of Endocrinology, Leiden University Medical Centre, 2333 ZA Leiden, The Netherlands

**Keywords:** pseudoxanthoma elasticum, vascular calcification, computed tomography, etidronate

## Abstract

**Background:** Pseudoxanthoma elasticum (PXE), a rare genetic disorder presenting with slowly progressing calcification of various tissues, including the arteries, is caused by mutations in the *ABCC6* gene that lead to the reduction of pyrophosphate, a natural inhibitor of calcification. We showed that, compared to a placebo, the cyclical administration of etidronate, a stable pyrophosphate analog, significantly reduced arterial calcification assessed by low-dose CT scans after one year. The aim of the present prospective, single center, observational cohort study was the assessment of the efficacy and safety of cyclical etidronate in patients treated for periods longer than one year. **Methods:** Seventy-three patients were followed for a median of 3.6 years without etidronate and 2.8 years with etidronate, and each patient served as their own control. **Results:** The median absolute yearly progression of total calcification volume during the period with etidronate (388 [83–838] µL) was significantly lower than that without etidronate (761 [362–1415] µL; *p* < 0.001). The rates of the relative progression of arterial calcification were 11.7% (95% CI: 9.6–13.9) without etidronate compared to 5.3% (95% CI: 3.7–7.0) with etidronate, after adjustment for confounders. **Conclusions:** The cyclical administration of etidronate for nearly 3 years significantly reduced the progression rate of arterial calcification in patients with PXE with pre-existing calcifications without any serious adverse effects.

## 1. Background

Pseudoxanthoma elasticum (PXE, OMIM#264800) is a rare autosomal recessive disorder involving the skin, the eyes and the cardiovascular system, characterized by the fragmentation and calcification of elastic fibers in affected tissues [1,2]. Vascular calcifications, predominantly in the intracranial arteries and the arteries of the legs, a hallmark of PXE, progress slowly, are particularly prominent after the fourth decade of life and are associated with increased risk of complications with a high morbidity, such as peripheral arterial disease and stroke [3,4,5]. There is currently no approved treatment for PXE. 

The pathogenesis of ectopic mineralization in pathologically altered tissue is still poorly understood [6]. Studies, however, of heritable ectopic mineralization disorders with defined gene defects have helped in the elucidation of mechanisms inducing ectopic mineralization, and PXE is the prototype of such disorders [7]. PXE is caused by loss-of-function mutations in the *ABCC6* gene [8], which codes for the ABCC6 transporter in the hepatocyte [9,10]. While the substrate of the ABCC6 transporter is not fully elucidated, the mutations identified in patients with PXE lead to the reduction of circulating pyrophosphate, a potent natural inhibitor of calcification, which has high affinity for calcium crystals and impairs their formation and dissolution in vitro and in vivo [9,11,12,13,14,15]. A stable pyrophosphate analog, bisphosphonate etidronate, had been previously approved in the USA and Europe as a daily treatment for patients with conditions associated with the ectopic calcification of soft tissues, such as that following total hip replacement or spinal cord injury [16]. Etidronate appeared, therefore, to be a potential therapeutic option for patients with PXE, a notion supported by studies of *Abcc6*^−/−^ mice, in which treatment with oral or subcutaneous etidronate reduced the appearance or the progression of calcifications [17,18]. However, etidronate doses required for the efficacious treatment of calcifications in animals and humans are high, and when given continuously for long periods may impair the normal mineralization of bone, causing osteomalacia, a reversible complication, after the discontinuation of treatment [16]. Notwithstanding, the pharmacological properties of bisphosphonates allow for the efficacious, safe administration of high doses intermittently, as has been repeatedly shown in the treatment of patients with osteoporosis [19]. 

We hypothesized earlier that a high dose of oral etidronate given intermittently to patients with PXE will safely reduce the ectopic vascular mineralization of patients with PXE, and we tested this hypothesis for the first time in a 1-year, randomized, double-blind, placebo-controlled clinical trial (TEMP trial). Compared to the placebo, the cyclical administration of etidronate (20 mg/kg/day for 2 weeks every 12 weeks) significantly reduced arterial calcification progression without important safety concerns [20,21]. Other studies in different populations, such as patients with kidney failure or diabetes, also show an inhibited growth of arterial calcification by etidronate [22,23,24,25,26], but the majority of studies had a treatment period no longer than 1 year. In one study in patients with end-stage kidney disease, it was shown that treatment with etidronate for 23 months prevented the further growth of arterial calcification [23].

PXE, however, is a chronic progressive disease requiring chronic treatment, and clinical studies of longer durations are needed to better define the role of etidronate in the management of patients with this rare disorder. The aim, therefore, of the present study was the assessment of the efficacy and safety of cyclical etidronate in patients with PXE treated for periods longer than one year.

## 2. Methods

### 2.1. Study Design

This was a prospective, observational, cohort study of patients with PXE diagnosed by the Plomp criteria [27] and followed at the UMC Utrecht Expertise Center for Pseudoxanthoma elasticum (UCEP). 

### 2.2. Patient Characteristics

Included in this study were patients ≥ 18 years with evidence of arterial calcification on a CT scan of the legs, routinely acquired in all patients during their first visit to our center, who received cyclical etidronate (20 mg/kg/day for 2 weeks every 12 weeks) for periods longer than one year. They were all participants of the Dutch PXE Registry, a prospective cohort study with pseudonymized data from regular care visits. Exclusion criteria were the same as those of the TEMP trial, namely, severe renal impairment, known abnormality of the esophagus, known sensitivity to etidronate, use of any bisphosphonate during the past 5 years, osteomalacia, chronic diarrhea, pregnancy, claustrophobia, hypocalcemia (serum calcium <2.20 mmol/L) and vitamin D deficiency (serum 25-OH vitamin D <35 nmol/L). 

### 2.3. Intervention and Processes

Eligible patients received etidronate for the first time either during the TEMP trial or later, as part of routine care, if they fulfilled the inclusion and exclusion criteria of the TEMP trial and they had a CT scan of the legs at least 6 months before starting etidronate therapy. Etidronate was provided in 400 mg tablets, and patients were instructed to refrain from eating or drinking anything other than water for two hours before and two hours after taking etidronate. The period without treatment always preceded that with treatment, and each patient served as her/his own control, ensuring a repeated measures design that also limits the impact of confounders. 

Etidronate was initially provided by Uni-Pharma Kleon Tsetis Pharmaceutical Laboratories SA (Athens, Greece), and later by Tiofarma B.V. (Oud-Beijerland, The Netherlands). Etidronate was produced for the patients with PXE in the UCEP, according to Good Manufacturing Guidelines (GMP). 

Arterial calcifications were assessed by low-dose CT scans as previously described [21]. In brief, non-contrast, thin-slice multi-detector CT scans were acquired on multiple scanners. The data were reconstructed at a slice thickness of 5 mm at increments of 4 mm, and a calcification was defined as an arterial wall lesion with a density over 130 Hounsfield Units (HUs), independent of their size. Arterial calcification volume, in microliters (µL), was measured using in-house-developed software (iX Viewer, version 1.0.0.0). Calcium volumes were corrected for vendor differences using correction factors established with a phantom study, described previously [4]. The total arterial calcification volume denotes the sum of calcifications of the intracranial carotid artery, the external carotid artery, the coronary artery, the aorta, the iliac artery and the leg artery. When calcification in a certain artery was not measurable (e.g., due to a stent) this artery was excluded from the total volume of all follow-up measurements of the patient.

For every visit in which a CT scan was performed, clinical information was obtained from medical records, and the closest available laboratory value was used, with specific attention being paid to risk factors for cardiovascular disease. The reported systolic and diastolic blood pressure (SBP and DBP) was the average of three consecutive measurements, smoking was categorized into current, former and never smoking and Body Mass Index (BMI) was defined as weight divided by height squared (kg/m^2^). Serum total cholesterol, high-density lipoprotein cholesterol (HDL-c), triglycerides, glucose, creatinine, phosphate and alkaline phosphatase were measured in the hospital laboratory with standard protocols. Low-density lipoprotein cholesterol (LDL-c) values were computed using the Friedewald formula. The estimated glomerular filtration rate (eGFR) was determined with the CKD-EPI formula. 

### 2.4. Statistical Analysis 

Normally distributed data are presented as means and standard deviations (SDs), non-normally distributed data as medians and interquartile ranges (IQRs) and categorical data as frequencies and percentages. Total arterial calcification without and with etidronate was measured in each individual patient, and the progression in each period was calculated as the absolute change in arterial calcification volume per year and compared for the two with the Mann–Whitney U test. In addition, the progression in calcification over time was calculated for every individual patient before and during treatment with the use of linear mixed models (LMMs). These models were further adjusted for potential clinical and medication confounders (gender, age, BMI, SBP, smoking status, lipid-lowering therapy, anti-thrombotic medication, glucose-lowering medication, antihypertensive medication and serum LDL-c). Statistical analyses were performed with R 4.2.3 (R Foundation for Statistical Computing, Vienna, Austria) and RStudio Desktop 2023.09.1 (Posit, Boston, MA, USA). A detailed description of the statistical methodology is given in the Supplementary Materials. 

## 3. Results

**Figure 1:**
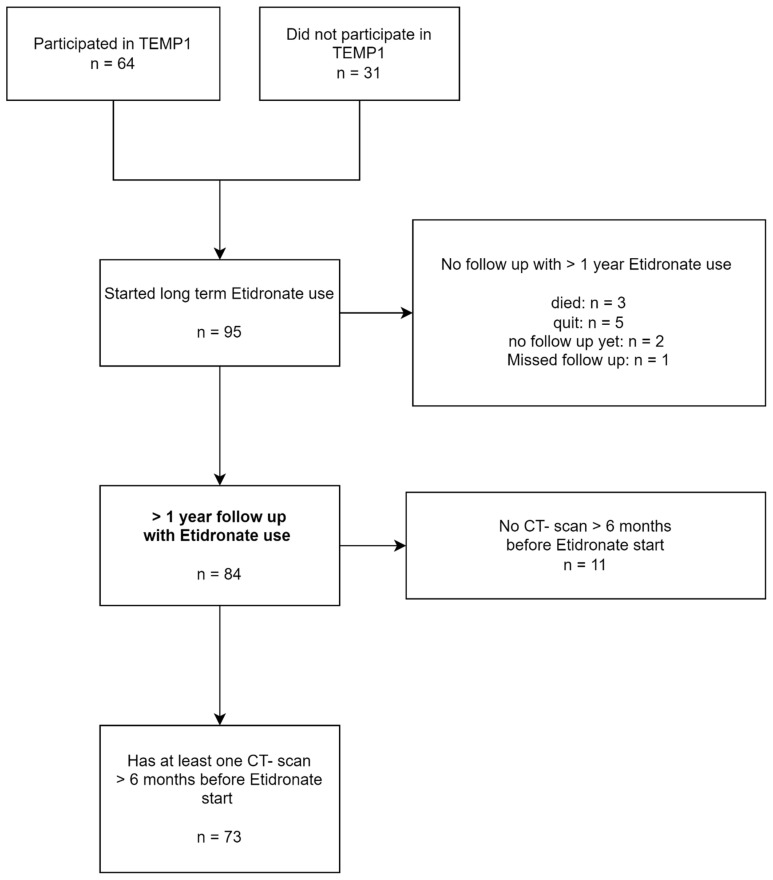
Flowchart of participants in the study; TEMP = Treatment of ectopic mineralization in Pseudoxanthoma elasticum.

Of the 95 bisphosphonate-treatment-naïve patients who met the inclusion criteria, 22 were excluded from the analysis (see flowchart in Figure 1) while all were included in the analysis of adverse effects of treatment. In a subgroup of treated patients (*n* = 20), the administration of etidronate was interrupted due to a temporary shortage of medication. Analyses included all 73 patients (intention-to-treat), and a separate analysis of efficacy was also performed for the 53 patients with uninterrupted treatment (per protocol). 

### 3.1. Baseline Characteristics

At baseline, the 73 patients had a mean age of 57 years, 45% were male and 10% had a history of a cerebrovascular events (Table 1). The median follow-up time without etidronate was 3.6 [IQR 1.3–4.2] years, and during this period a total of 243 CT scans were performed. The median follow-up time on etidronate was 2.8 [IQR 2.6–4.7] years, with 134 CT scans performed in that period. Between the baseline and the start of etidronate therapy, there had been an increase in the use of antihypertensive and lipid-lowering medications, reflecting the guidance for the cardiovascular risk management of patients with PXE at the UCEP. During this period, median LDL-c decreased from 3.1 [IQR 2.3–3.7] mmol/L to 2.2 [IQR 1.8–2.7] mmol/L, while other cardiovascular risk factors, such as BMI, SBP and eGFR, did not change. 

### 3.2. Progression of Arterial Calcification 

**Figure 2:**
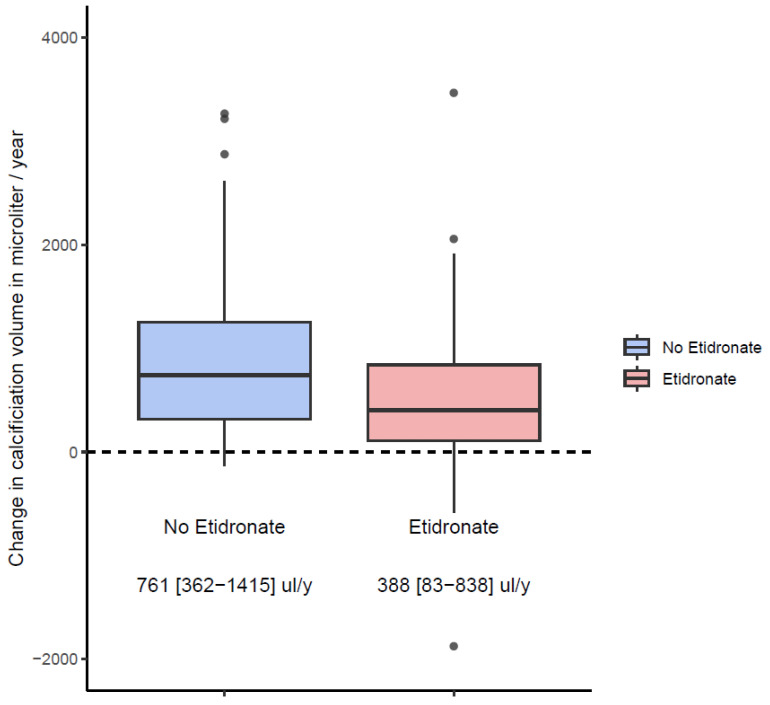
Boxplots depict the absolute change in arterial calcification volume per year when subjects were not on etidronate (blue), and when they were on etidronate (red), *p* < 0.001 (statistical analysis with Mann–Whitney U test).

**Figure 3:**
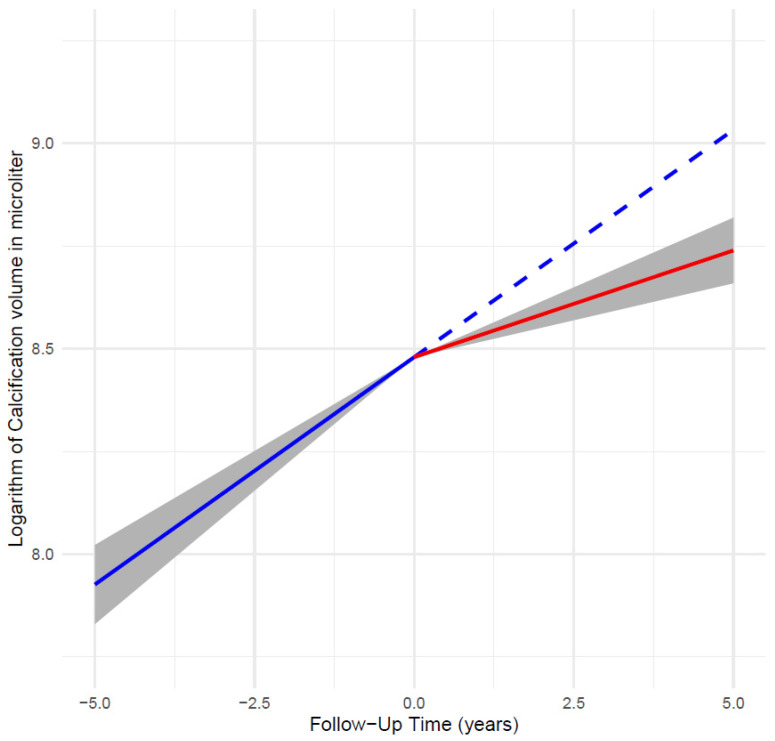
Progression of arterial calcification shown as the estimated trajectories of log arterial calcification volume without etidronate (blue) and with etidronate (red), based on LMM (linear mixed model), with 95% CI around model estimate (gray). Dashed line is the extrapolated trajectories.

The median increase in total calcification volume in the period without etidronate was 761 [362–1415] µL per year and decreased significantly to 388 [83–838] µL per year in the period with etidronate (*p* < 0.001; Figure 2). There was no significant interaction between baseline age and calcification progression either without or with etidronate (*p* = 0.59 and *p* = 0.10, respectively). The relative annual progression of arterial calcification determined with the LMM was 12.0% (95% CI: 10.2–13.8) without etidronate and 5.2% (95% CI: 3.7–6.8) with etidronate, a reduction in progression rate of 57%. After adjustment for baseline age, progression rates remained the same at 11.9% (95% CI: 10.1–13.8) and 5.3% (95% CI: 3.8–6.8) per year. Further adjustment for all potential confounders, including lipid-lowering therapy and statin therapy that could have affected calcification, did not change the progression rates that remained practically the same at 11.7% (95% CI: 9.6–13.9) and 5.3% (95% CI: 3.7–7.0) per year for the two periods, respectively, with a reduction rate of 55% (Figure 3, Table 2). There were no age and gender differences in progression rates that were also similar across tertiles of calcium scores (Supplementary Tables S1–S3).

In the 53 patients with no treatment interruption (per protocol analysis), the progression rate of total arterial calcification volume was 11.6% (95% CI 9.0–14.1%) without etidronate and 3.9% (95% CI: 1.0–7.2%) with etidronate, adjusted for confounders. 

### 3.3. Adverse Events 

No serious adverse events related to etidronate were reported. Three patients died (pulmonary carcinoma, cerebrovascular hemorrhage and vascular dementia due to multiple cerebrovascular infarctions) and another three patients sustained traumatic fractures. Serum phosphate concentrations and alkaline phosphatase activity, measured for 71 and 61 patients, respectively, in blood obtained at least two weeks after the end of the last dose of etidronate, were within their respective normal ranges: median phosphate 1.1 mmol/L (range 0.6–1.5) and median alkaline phosphatase activity 68.5 U/L (range 39–114). There were no incidences of osteonecrosis of the jaw or atypical fractures in the whole cohort during the entire period of observation. Gastrointestinal complaints were the most common side effects of long-term treatment, particularly abdominal cramps or pain and diarrhea in 19% and 17% of patients, respectively. In two patients, treatment was stopped because of such complaints, while in three patients, lowering the dose of etidronate improved the symptoms. 

## 4. Discussion

We show here that cyclical etidronate given to patients with PXE for a median period of about 3 years significantly reduced the progression of their arterial calcifications independently of potential confounders of cardiovascular risk. This study confirms and extends our one-year placebo-controlled assessment of the efficacy of this high-dose etidronate given intermittently to patients with PXE in the TEMP trial [20,21]. Differently, however, from TEMP, the present study was observational but had certain unique features. Firstly, the set up at the UCEP ensured the standard clinical, laboratory and radiological follow-up common for all studied patients with PXE. Secondly, the design allowed for the selection of all patients as their own controls. Thirdly, the long-term follow-up was for a median period longer than 6 years, and, finally, the relatively frequent assessment of vascular calcifications by low-dose CT scans (a total of 377 or 5 scans per patient). 

Arterial calcification, independently of cardiovascular risk factors, is associated with increased risk of cardiovascular morbidity and mortality [28,29,30] and is a major disease manifestation of PXE [31] that contributes to the higher risk of peripheral artery disease [5] and cerebrovascular disease [3,32]. The arterial calcification, as measured by CT scan imaging, is currently the most accurate indicator of vascular disease severity in PXE [4,31], and interventions that decrease it may, therefore, lower the risk of cardiovascular disease in these patients. Given the slow progression and the rarity of the disease, we believe that a controlled intervention study with hard endpoints can never be performed. However, the highly significant association of CT scan findings with the clinical severity of the disease strongly supports the use of the technique as a surrogate efficacy endpoint of any intervention study. 

The efficacy results of the present study, the longest controlled intervention study performed in patients with PXE, not only supports the short-term results of the TEMP trial but also demonstrates persistence of the positive therapeutic outcome. However, while in both the TEMP trial and in the present study, the total arterial calcification mass increased in untreated patients by 6.3% and 11.4% per year, respectively, progression was halted in treated patients in the TEMP group, while in the present study, some progression in arterial calcification was measured. This apparent discrepancy may be, at least, partly explained by the temporary interruption of treatment in a subset of patients, because in the per protocol analysis, a lower rate was measured. Interestingly, the total median change in arterial calcification (no treatment minus treatment) in both studies was practically the same: 8.7% and 8%, respectively. 

In the present study, no serious adverse events related to etidronate were observed, and the frequency and nature of the reported side effects were in line with those expected and previously recognized from treatment with etidronate [16]. These were primarily gastrointestinal complaints that led to the discontinuation of therapy in two patients. while in three, the reduction in the dose eliminated the problem. Furthermore, no cases of osteonecrosis of the jaw or atypical fractures were reported. Importantly, at the end of the treatment period, serum phosphate concentrations and alkaline phosphatase values were normal in all patients in whom these were measured. There was, thus, no evidence for concern about the safety of treatment on bone and mineral metabolism. These results are reassuring because a controlled study of similar length with the etidronate dose used here has never been previously reported for any indication. 

Despite the already discussed strengths of the present study, the lack of a placebo group remains a limitation, but the planning of such a study with similar length as the present one is highly unlikely. In addition, the results may not be applicable to patients without already measurable calcifications. Finally, clinically relevant measures, such as quality of life parameters, were not recorded in the present study due to its observational design and their dependence on factors other than etidronate (e.g., aging), resulting in a higher risk of bias. We hope that a currently ongoing RCT of younger patients with PXE and no manifest cardiovascular disease (TEMP-PREVENT trial) [33] will answer some of these questions.

## 5. Conclusions

Cyclical etidronate administered to patients with PXE in the regimen used here is safe and associated with a reduced progression of arterial calcification in adult patients with PXE with pre-existing calcifications. These findings, together with the favorable results of the TEMP clinical trial, support the aim to repurpose etidronate for the treatment of patients with PXE, for which there is already interest. However, due to the limitations of the present study, additional studies, such as the currently ongoing evaluation of the efficacy and safety of etidronate in younger patients, are necessary.

## Figures and Tables

**Table 1:** Patient characteristics.

	Baseline	Start of Etidronate	End of Follow-Up
Time since baseline, years	0	3.6 [1.3–4.2]	6.8 [6.2–7.2]
Age, years	57.3 (7.8)	60.2 (8.1)	63.8 (7.7)
Male sex, *n* (%)	33 (45)	33 (45)	33 (45)
History of CV event, *n* (%)			
Cardiac event	2 (3)	2 (3)	2 (3)
Cerebrovascular event	7 (10)	11 (15)	14 (19)
Peripheral vascular event	3 (4)	3 (4)	3 (4)
Multiple events	3 (4)	3 (4)	3 (4)
None	58 (80)	54 (74)	51 (70)
Smoking status, *n* (%)			
Current smoker	7 (10)	7 (10)	5 (7)
Former smoker	36 (49)	36 (49)	38 (52)
Never smoker	30 (41)	30 (41)	30 (41)
Glucose-lowering medication, *n* (%)	2 (3)	3 (4)	4 (6)
Blood pressure-lowering medication, *n* (%)	10 (14)	17 (23)	25 (34)
Lipid-lowering medication, *n* (%)	31 (43)	50 (69)	58 (80)
Antithrombotic medication, *n* (%)	15 (21)	18 (25)	20 (27)
Diabetes Mellitus type 1, *n* (%)	1 (1)	1 (1)	1 (1)
Diabetes Mellitus type 2, *n* (%)	1 (1)	4 (6)	4 (6)
BMI, kg/m^2^	25.7 (3.4)	25.8 (3.6)	26.3 (3.7)
Systolic blood pressure, mmHg	142 [130–153]	141 [131–154]	146 [134–157]
Diastolic blood pressure, mmHg	80 [75–87]	79 [73–87]	82 [76–88]
Total cholesterol, mmol/L	5.5 [4.3–6.3]	4.2 [3.7–4.8]	4.0 [3.7–4.8]
Triglycerides, mmol/L	1.1 [0.9–1.6]	1.0 [0.8–1.4]	1.0 [0.7–1.3]
HDL-c, mmol/L			
Men	1.3 [1.3–1.5]	1.3 [1.3–1.6]	1.3 [1.3–1.6]
Women	1.7 [1.5–1.9]	1.7 [1.4–2.0]	1.7 [1.4–2.0]
LDL-c, mmol/L	3.1 [2.3–4.1]	2.2 [1.9–2.8]	2 [1.7–2.7]
eGFR, mL/min/1.73 m^2^	93 [80–100]	93 [81–100]	88 [78–96]
Total calcification volume, µL	6245 [2268–10,010]	9677 [3108–13,693]	10,482 [4201–15,308]

Data are presented as means (SDs) or medians [IQRs]. If the data are presented as n (%), this is depicted in the table. CV = cardiovascular, BMI = Body Mass Index, HDL-c = high-density lipoprotein cholesterol, LDL-c = low-density lipoprotein cholesterol, eGFR = estimated glomerular filtration rate.

**Table 2:** Calcification of annual progression rate in periods without and with etidronate.

	Model 1 Annual Progression Rate	Model 2 Annual Progression Rate	Model 3 Annual Progression Rate
Progression without etidronate, % (95% CI)	12.0 (10.2–13.8)	11.9 (10.1–13.8)	11.7 (9.6–13.9)
Progression with etidronate, % (95% CI)	5.2 (3.7–6.8)	5.3 (3.8–6.8)	5.3 (3.7–7.0)
Relative difference, %	−57	−55	−55

Annual progression rates derived from linear mixed model (LMM) 1, 2 and 3. Model 1: follow-up time and interaction term etidronate * follow-up time. Model 2: model 1 and additional adjustment for age at baseline using natural splines. Model 3: model 2 and additional adjustment for lipid-lowering medication, anti-thrombotic medication, glucose-lowering medication, blood pressure-lowering medication, LDL-c, BMI, SBP and smoking status. Annual progression is calculated by exponentiating estimates from the LMM, subtracting 1 and multiplying by 100.

## Data Availability

The data can be made available upon request.

## Supplementary Materials

The following supporting information can be downloaded at https://www.mdpi.com/article/10.3390/jcm13164612/s1: Supplementary Methodology: Statistical analysis. Table S1: Results stratified for sex. Table S2: Results stratified for tertiles of baseline calcification volume. Table S3: Results stratified for tertiles of age at baseline. Figure S1. Individual progression of arterial calcification volume (log transformed), without etidronate (blue) and during etidronate (red).

## Abbreviations

PXE	Pseudoxanthoma elasticum
ABCC6	ATP-binding cassette sub-family C member 6
PPi	Inorganic pyrophosphate
RCT	Randomized controlled trial
CT	Computed tomography
UMC	University Medical Center
UECP	UMC Utrecht Expertise Center for PXE
BMI	Body Mass Index
SD	Standard deviation
IQR	Interquartile range
HUs	Hounsfield Units
LDL-c	Low-density lipoprotein cholesterol
HDL-c	High-density lipoprotein cholesterol
eGFR	Estimated glomerular filtration rate
CKD-EPI	Chronic Kidney Disease Epidemiology Collaboration
SBP	Systolic blood pressure
DBP	Diastolic blood pressure
TEMP	Treatment of ectopic mineralization in Pseudoxanthoma elasticum
CI	Confidence Interval
LMMs	Linear mixed models
