# Supplementary material for: Cyclical Etidronate Reduces the Progression of Arterial Calcifications in Patients with Pseudoxanthoma Elasticum: A 6-Year Prospective Observational Study
Source: J Clin Med. 2024 Aug 7;13(16):4612. doi: 10.3390/jcm13164612 (PMC11354836; doi:10.3390/jcm13164612)
Supplement: Supplementary file 1 [file jcm-13-04612-s001.zip › jcm-3064134-supplementary.pdf]

Supplementary Methods: Statistical Analysis

To assess the effect of etidronate on the progression of total arterial calcification, we compared the progression in the period without etidronate to the progression in the period with etidronate. Firstly, the absolute annual change in total arterial calcification was calculated. For each period the last calcium score was subtracted from the initial calcium score and divided by the time difference in years. Secondly, the relative annual change was calculated for the period without etidronate and the period with etidronate. The absolute difference was divided by the first calcium score of the period times 100 and divided by the time difference in years. The absolute annual change in total arterial calcification was compared for the period without etidronate to the period with etidronate using a Mann-Whitney U test.

Additionally, the association of etidronate with the progression of arterial calcification was assessed with the use of linear mixed models (LMM). A LMM was built with the natural logarithm of arterial calcification volume (plus 1 to deal with zero values) as outcome variable, and follow-up time, etidronate use. Etidronate use was added as main effect and as interaction term with follow-up time. Random intercepts and slopes for follow-up time were modelled for every PXE-patient. An additional model was built where baseline age was added as a main effect. Baseline age was modelled as a natural spine with 3 knots, which significantly improved our model fit, based on Akaike Information Criterion (AIC). Lastly, a third model was built which included the following time-varying potential confounders as determinants: lipid-lowering therapy, anti-thrombotic medication, glucose-lowering medication, antihypertensive medication, LDL-c, BMI, SBP and smoking status. For reader comprehension, we further divided age and calcification volume in tertiles. Additionally, we performed stratification based on sex.

Because total arterial calcification was log-transformed, due to the known exponential growth [4], only a relative progression rate on a non-logarithmic scale can be interpreted. Therefore, we present the relative progression rate as an annual percentage increase in total arterial calcification. To calculate the annual progression rate during the period without etidronate, the estimate for the 'follow-up time' term in the LMM was exponentiated, subtracted by one, and then multiplied by 100. The same calculation was performed on the limits of the 95% confidence interval (CI) surrounding the estimate for follow-up time. In order to calculate the progression rate of the period with etidronate, a combined estimate was computed: the sum of the estimate for follow-up time and the interaction term of follow-up time with etidronate. The 95% CI of the combined estimate was calculated with the use of estimated marginal means. The combined estimate, and the limits of the 95% CI were also exponentiated and expressed as a percentage by subtracting one and multiplying by 100.

Supplementary Table S1. Annual progression rate in the periods with and without etidronate stratified for sex.

	Men (n = 33)	Women (n = 40)	P for interaction
Annual progression rate without etidronate, % (95% CI)	10.8 (8.7 – 13.0)	12.6 (9.9 – 15.4)	0.85
Annual progression rate with etidronate, % (95% CI)	5.3 (3.7 – 7.0)	5.1 (2.6 – 7.5)	0.23
Relative difference in progression rate	-51%	-40 %	

Annual progression rates derived from linear mixed model with follow-up time, interaction term for follow up stratified for sex, adjusted for baseline age. Annual progression is calculated by exponentiating estimates from the LMM, subtracting 1 and multiplying by 100.

Supplementary Table S2. Annual progression rate in the periods with and without etidronate stratified for baseline calcification volume.

	Tertile 1 (n = 24)<br>63 – 3334 µL	Tertile 2 (n = 24)<br>3334 – 8164 µL	Tertile 3 (n = 25)<br>8164 – 54905 µL
Annual progression rate without etidronate, % (95% CI)	14.0 (10.9 – 17.2)	13.0 (7.9 – 19.4)	8.1 (2.8 – 13.7)
Annual progression rate with etidronate, % (95% CI)	7.3 (5.0 – 9.8)	3.5 (0.8 – 6.2)	4.7 (2.3 – 7.3)
Relative difference in progression rate	-48 %	- 70%	- 42 %

Annual progression rates derived from linear mixed model with follow-up time, interaction term for follow up time and adjusted for baseline age with spline, stratified for tertile of baseline calcification. Annual progression is calculated by exponentiating

Supplementary Table 3. Annual progression rate in the periods with and without etidronate stratified for age at baseline.

	Tertile 1 (n = 24) 39.7 – 58.4 years	Tertile 2 (n = 24) 58.4 – 64.5 years	Tertile 3 (n = 25) 64.6 – 79.5 years	P for interaction
Annual progression rate without etidronate, % (95% CI)	14.6 (11.4 – 17.9)	10.0 (4.5 – 15.9)	10.7 (5.3 – 16.5)	0.33
Annual progression rate with etidronate, % (95% CI)	5.6 (3.2 – 8.0)	4.1 (1.7 – 6.7)	6.4 (3.6 – 9.2)	0.67
Relative difference in progression rate	-62 %	-59 %	-40 %	

Annual progression rates derived from linear mixed model with follow-up time, interaction term for follow up time and adjusted for baseline age with spline, stratified for tertile of age at baseline. Annual progression is calculated by exponentiating estimates from the LMM, subtracting 1 and

Supplementary Figure S1

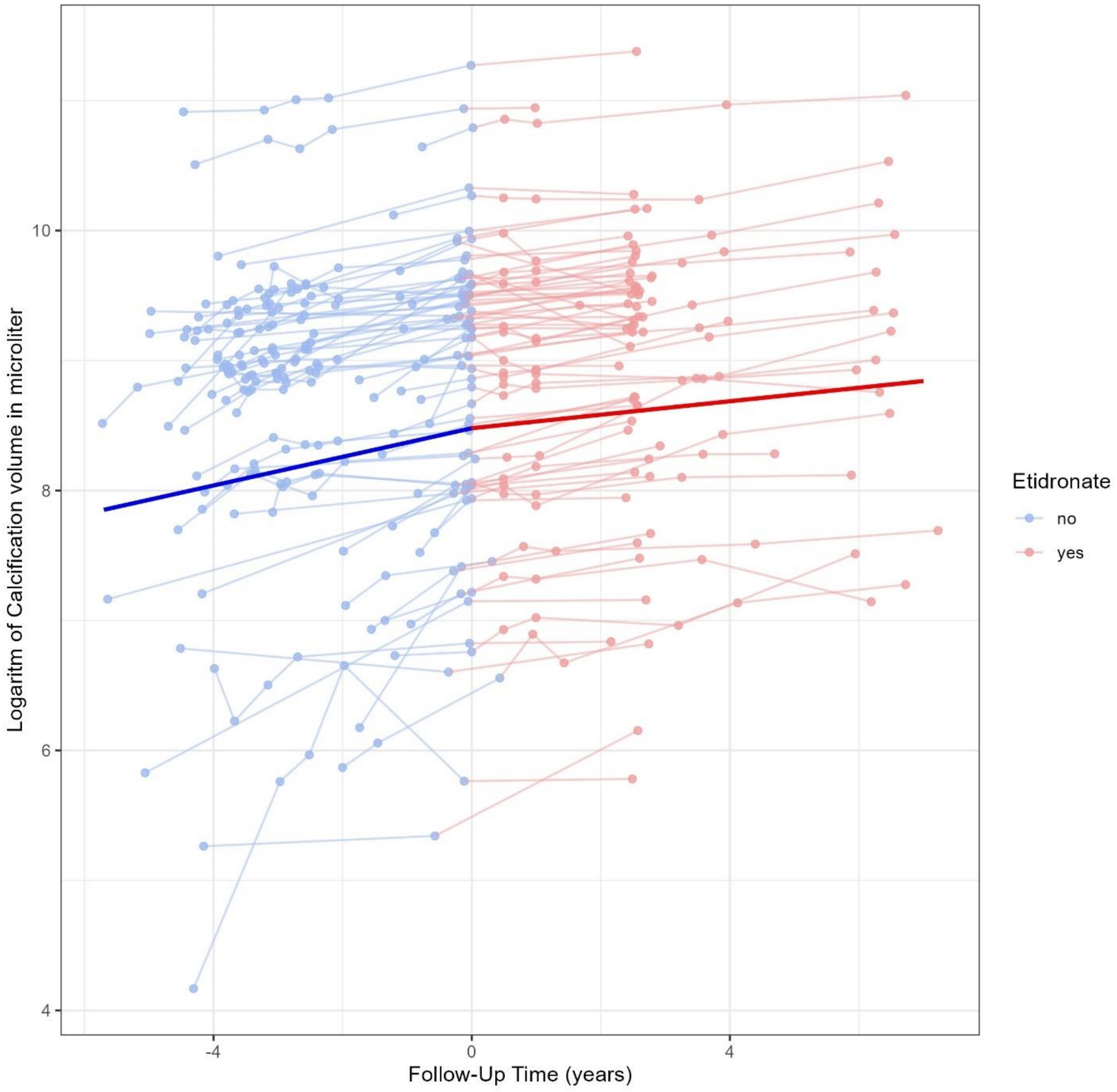

Supplementary Figure S1. Individual progression of arterial calcification volume (log transformed), without etidronate (blue) and during etidronate (red). Each dot represents a CT scan. Bold lines represent the results from the mixed model.
